# Europäische Perspektiven auf den Arbeits- und Gesundheitsschutz: Impulse für eine globaler orientierte Arbeitswissenschaft

**DOI:** 10.1007/s41449-021-00251-0

**Published:** 2021-05-20

**Authors:** Armin Windel, Sebastian Haus-Rybicki

**Affiliations:** Bundesanstalt für Arbeitsschutz und Arbeitsmedizin, Friedrich-Henkel-Weg 1–25, 44149 Dortmund, Deutschland

**Keywords:** Europäische Union, Digitalisierung, Künstliche Intelligenz, Vulnerable Beschäftigte, Arbeitsbedingte Erkrankungen, Arbeitsschutzsysteme, European Union, Digitalisation, AI, Vulnerable workers, Work-related diseases, OSH systems

## Abstract

In den vergangenen Jahrzehnten hat sich die Sicherheit und Gesundheit der Erwerbstätigen in Europa stetig verbessert, was nicht zuletzt auf die Umsetzung arbeitswissenschaftlicher Erkenntnisse zurückzuführen ist. Gleichwohl stehen die europäischen Arbeitsschutzsysteme vor einer Reihe von Herausforderungen, die nicht zuletzt im Zuge der Covid-19-Pandemie verstärkt in das Bewusstsein der Politik und der Öffentlichkeit gerückt sind. Der Beitrag thematisiert beispielhaft einige dieser zentralen Herausforderungen des Arbeitsschutzes aus einer europäischen Perspektive. Grundlage ist ein Positionspapier, das unter Federführung der Bundesanstalt für Arbeitsschutz und Arbeitsmedizin (BAuA) gemeinsam mit den führenden europäischen Arbeitsschutzinstituten aus dem Forschungsnetzwerk PEROSH anlässlich des Konsultationsprozesses der EU-Kommission für eine neue europäische Arbeitsschutzstrategie entstanden ist. In diesem Beitrag geht es dabei nicht nur darum, die europäische Dimension vieler Herausforderungen herauszustellen. Vielmehr skizziert er diese auch als strategische Handlungsfelder, um die Potenziale der Arbeitswissenschaft bezüglich der Erarbeitung gemeinsamer europäischer Lösungsansätze – als Grundlage für die Lösung von Problemen der Globalisierung – hervorzuheben.

## Abstract (en)

In recent decades, the safety and health of workers in Europe has steadily improved, in no small part due to the implementation of the results of ergonomics research. However, European OSH systems are facing a number of challenges, which have been increasingly brought to the attention of policymakers and the public in the wake of the Covid-19 pandemic. This article examines some of these central challenges to occupational safety and health from a European perspective. It is based on a position paper prepared by the German Federal Institute for Occupational Safety and Health (BAuA) together with other European OSH institutes from the research network PEROSH on the occasion of the EU Commission’s consultation process for a new European OSH strategy. The article not only highlights the European specifics of many challenges. Rather, it also understands these challenges as strategic fields of action for ergonomics, in order to demonstrate the potential of ergonomics in the development of joint European OSH solutions—as basis for the solution of problems caused by globalisation.

## Zunehmende Komplexität und Dynamik der Arbeitswelt als Auftrag an anwendungsorientierte Wissenschaftsdisziplinen

Das deutsche Grundgesetz schützt in Artikel 5 die Freiheit von Wissenschaft, Forschung und Lehre, die sich unabhängig von staatlichen Einflüssen entwickeln können sollen.

Gleichzeitig ist die Wissenschaft zunehmend gefragt, sich mit gesellschaftlich relevanten Fragestellungen zu befassen und ihre Erkenntnisse so darzustellen, dass sie in die gesellschaftliche Debatte eingebracht werden können. Angesichts der hohen Bedeutung der Arbeit in der Gesellschaft, der sich verändernden Arbeitsbedingungen und des kontinuierlichen technologischen Wandels gilt dies insbesondere für die in der Arbeitswissenschaft versammelten Fachdisziplinen. Die hohe Anwendungsorientierung der Arbeitswissenschaft ist sowohl Anspruch als auch Verantwortung, Antworten auf die komplexen Herausforderungen einer zunehmend globalisierten Welt zu finden. Dabei ist arbeitswissenschaftliche Forschung selbstverständlich international: Erkenntnisse zu den relevanten Forschungsfragen unserer Zeit finden sich natürlich nicht nur innerhalb der deutschsprachigen Wissenschaftscommunity. Aus einer solchen Perspektive hat arbeitswissenschaftliche Forschung den Auftrag, sich mit gesellschaftlichen Herausforderungen der zunehmend globalisierten Welt zu befassen, wie etwa den Auswirkungen der zunehmenden Digitalisierung, dem Kampf gegen Krankheiten oder der Schaffung guter Arbeitsbedingungen für alle Erwerbstätigen. Hierzu kann die Arbeitswissenschaft wissenschaftliche Fundierung und Lösungsansätze beitragen. Angesichts der weit vorangeschrittenen europäischen Integration bildet die Europäische Union, das heißt genauer ihre Institutionen, Prozesse und politischen Initiativen, einen außerordentlich wichtigen Bezugsrahmen für die Umsetzung arbeitswissenschaftlicher Erkenntnisse.

Der vorliegende Beitrag will die Arbeitswissenschaft dazu anregen, die Politik der EU, insbesondere im Bereich von Sicherheit und Gesundheit bei der Arbeit, verstärkt in den Blick zu nehmen, um hieraus Impulse für das Selbstverständnis und das eigene Forschungshandeln, aber auch eine verstärkte Internationalisierung, abzuleiten. Selbstverständlich gehen wir davon aus, dass Arbeitswissenschaftlerinnen und Arbeitswissenschaftler in der Bearbeitung konkreter Forschungsfragen immer auch international ausgerichtet sind, etwa dadurch, dass sie in englischsprachigen Journals veröffentlichen oder den wissenschaftlichen Diskurs auf internationalen Konferenzen suchen. Gleichzeitig weist ein kritischer Überblick über die Beiträge der letzten GfA-Konferenzen darauf hin, dass Forschungsfragen, die sich durch eine europäisch-vergleichende Perspektive auszeichnen und zur Lösung gemeinsamer europäischer Herausforderungen beitragen wollen, stärker als bislang adressiert werden könnten. Hierzu zählen auch methodische Forschungsfragen, die unterschiedliche Interventionsansätze in den Mitgliedstaaten betrachten oder auf eine vergleichende Evaluation von länderspezifischen Präventionsstrategien abzielen.

## Eine neue Arbeitsschutzstrategie der EU als Handlungsrahmen für angewandte Forschung

Ausgangspunkt des Beitrags ist die neue Arbeitsschutzstrategie der EU 2021-2027, die sich aktuell in Vorbereitung befindet und im zweiten Quartal 2021 vorgestellt wird. Der Rat der Europäischen Union und die EU-Kommission betrachten den neuen Strategischen Rahmen für Gesundheit und Sicherheit am Arbeitsplatz 2021-2027 als einen von mehreren Beiträgen zur Umsetzung der Europäischen Säule Sozialer Rechte (ESSR). Die ESSR begreift unter anderem die Vereinbarkeit von Berufs- und Privatleben sowie ein gesundes, sicheres und gut angepasstes Arbeitsumfeld als ein Recht aller EU-Bürgerinnen und EU-Bürger.

Ein weiteres Instrument der EU zur Umsetzung der gemeinsamen politischen Ziele wurde mit Beginn dieses Jahres neu aufgelegt: Als Spiegel der gesellschaftlichen und wirtschaftlichen Herausforderungen verfolgt auch das Forschungsförderprogramm „Horizon Europe“ das Ziel der Förderung der Zusammenarbeit zwischen den Mitgliedstaaten. In Fortsetzung des erfolgreichen Vorgängerprogramms „Horizon 2020“ werden in der zentralen Säule „Global Challenges and European Industrial Competitiveness“ auch Kernthemen der Arbeitswissenschaft adressiert (z. B. in den Clustern „Digitalisierung“ und „Gesundheit“).

Gemeinsam mit 12 weiteren europäischen Arbeitsschutzinstituten hat die BAuA ein Positionspapier verfasst und aktiv in den Konsultationsprozess der EU für die neue Arbeitsschutzstrategie eingebracht. Das Papier wurde im Rahmen der seit 2003 bestehenden „Partnership for European Research in Occupational Safety and Health“ (PEROSH) verfasst. Ihr gehören die führenden nationalen Arbeitsschutzinstitute aus derzeit 13 europäischen Ländern an. Anliegen der Partnerschaft ist unter anderem die Zusammenarbeit in Forschungs- und Entwicklungsprojekten sowie die Vertretung der Perspektive der Arbeitsschutzforschung gegenüber den europäischen Institutionen. Das PEROSH-Positionspapier zielt darauf ab, den Beitrag der angewandten Forschung zu den Herausforderungen für die Sicherheit und Gesundheit bei der Arbeit und zum europäischen Arbeitsschutzhandeln zu unterstreichen und die Forschungsperspektive stärker als bislang zu einem Bestandteil der europäischen Arbeitsschutzstrategie zu machen (PEROSH 2020).

## Europäische Herausforderungen für die Sicherheit und Gesundheit am Arbeitsplatz – Handlungsfelder für die Arbeitswissenschaft

Im Positionspapier für die EU-Kommission identifiziert PEROSH insgesamt neun zentrale Herausforderungen für die kommenden Jahre: Von den Risiken und Potenzialen der Digitalisierung und Künstlichen Intelligenz (KI) über die Prävention in spezifischen Beschäftigtengruppen bis hin zum Umgang mit Gefahrstoffen. Hinsichtlich dieser Herausforderungen wird nicht nur politischer Handlungsbedarf deutlich gemacht. Vielmehr sind sie auch als Handlungsfelder für die Forschung zu verstehen, da sie auch den Beitrag der Forschung zu den Zielen der europäischen Arbeitsschutzstrategie skizzieren. Zur Reflexion des anwendungsorientierten Forschungshandelns werden die für die Arbeitswissenschaft zentralen Themen nachfolgend im Überblick dargestellt. Hierbei wird versucht, den Mehrwert einer europäischen Perspektive anzureißen.

### Menschengerechte Gestaltung von KI-Systemen

Digitalisierung, Künstliche Intelligenz (KI) und fortschrittliche Robotik bestimmen seit einigen Jahren die nationale und internationale Debatte über die Zukunft der Arbeit (BMAS 2017; ILO 2019). Die Auswirkungen des technologischen Wandels auf die Wertschöpfung und Produktion, auf die Arbeitsprozesse, Arbeitsaufgaben und Arbeitsmittel sowie nicht zuletzt die Qualifikationserfordernisse werden teilweise als massiv eingeschätzt (EU-OSHA 2018). Neue Technologien verändern die Aufgabenverteilung zwischen Mensch und Technik, die auch aus einer ethischen Perspektive betrachtet werden muss. Digitalisierung und KI wirken sich auf die Tätigkeiten aus, die Beschäftigte im Rahmen ihrer Arbeit übernehmen, und verändern die Anforderungen, die an sie gestellt werden. Diese Entwicklungen haben auch weitreichende Auswirkungen auf die Sicherheit und Gesundheit bei der Arbeit. Sie stellen daher eine der zentralen Herausforderungen für die menschengerechte Gestaltung der Arbeit dar (Rothe et al. 2019).

Auch die Institutionen der Europäischen Union haben diese Herausforderung anerkannt. Auf EU-Ebene wird bereits seit einiger Zeit über die Sicherheit neuer digitaler Technologien diskutiert. Im Rahmen ihrer Digitalisierungsstrategie (EU KOM 2020a) lotet die EU-Kommission parallel zu Fragen der Technologie- und Innovationsförderung auch die regulatorischen Erfordernisse aus, die sich insbesondere hinsichtlich KI-Anwendungen und fortschrittlicher Robotik stellen. Anfang 2020 hat die Kommission beispielsweise in ihrem „Weißbuch zur Künstlichen Intelligenz“ Überlegungen zu einer einheitlichen europaweiten Regulierung potenzieller Risiken von KI-Systemen vorgestellt (EU KOM 2020b). Die Kommission thematisiert darin Fragen der Produkt- und IT-Sicherheit, der Schadenshaftung, des Datenschutzes, der Transparenz und des Vertrauens sowie nicht zuletzt der menschlichen Kontrolle und Aufsicht. Für das zweite Quartal 2021 hat die EU-Kommission einen horizontalen Rechtsakt zu KI gemeinsam mit der Novellierung der Maschinenverordnung in Aussicht gestellt. Die Nähe dieser Themen zu den Gestaltungsgrundsätzen der Arbeitswissenschaft ist offenkundig – trotz teils unterschiedlicher Begrifflichkeit.

Aus der Sicht von PEROSH liegen in diesem Handlungsfeld große Chancen für die anwendungsorientierte Arbeitsforschung, vor allem, weil noch viele Fragen hinsichtlich der Auswirkungen digitaler Technologien auf Sicherheit und Gesundheit ungeklärt sind. Um diese Fragen anzugehen, bietet z. B. die europäische Forschungsförderung in den kommenden vielversprechenden Möglichkeiten.

Im neuen Forschungsrahmenprogramm „Horizon Europe“ werden von der Forschung nicht nur Beiträge zur Sicherung der „technologischen Souveränität“, „strategischen Autonomie“ und „globalen industriellen Führerschaft“ Europas erwartet, sondern auch zur einer „menschen-zentrierten und ethischen Entwicklung und Nutzung neuer Technologien“. Im ersten Arbeitsprogramm 2021–22 für das Cluster „Digital, Industry and Space“ spielen somit auch arbeitswissenschaftliche Perspektiven wie die Arbeitsgestaltung, die Mensch-Technik-Interaktion, Gesundheitsrisiken sowie die Produktions- und Produktsicherheit eine große Rolle.

### Gute Arbeit für vulnerable Beschäftigtengruppen

Seit einigen Jahren führen ökonomische und gesellschaftliche Veränderungsprozesse weltweit zu einer größeren Vielfalt und Heterogenität in der erwerbstätigen Bevölkerung. Blickt man etwa auf die Beschäftigungsverhältnisse in Europa, so lässt sich ein Anstieg verschiedener Formen von atypischer Beschäftigung feststellen (Eurofound 2020). Atypische Beschäftigung definiert sich grundsätzlich durch die Abweichung vom Normalarbeitsverhältnis, wobei die unter dieses Merkmal fallenden Beschäftigungsverhältnisse ein breites und heterogenes Spektrum umfassen: Beispiele für atypische Beschäftigung sind Leih- oder Zeitarbeit, befristete Beschäftigung, Teilzeitarbeit, (Solo‑)Selbstständigkeit oder Mehrfachbeschäftigung. Im europäischen Verständnis fallen darunter auch „on-call work“, „casual work“ oder „seasonal work“, die im Zusammenhang mit der innerhalb Europas zunehmenden Arbeitsmobilität steht (Europäisches Parlament 2010; Eurofound 2019). Bei „Crowdwork“ und „Gigwork“ handelt es sich um neue Formen der Arbeitsorganisation durch Digitalisierung, die auch in herkömmlichen Arbeitsverhältnissen zu finden sind, sich aber besonders bei atypischen Beschäftigten zeigen.

Sowohl in der politischen als auch in der wissenschaftlichen Debatte ist umstritten, ob es sich bei atypischen Beschäftigungsformen um prekäre Beschäftigungen handelt und ob sie zu gesundheitlichen Beeinträchtigungen führen, beispielsweise hinsichtlich der Dauer der Befristung. Aus der Perspektive von PEROSH sollte arbeitswissenschaftliche Forschung unter Berücksichtigung der vorzufindenden Vielfalt der Arbeitsformen in Europa für die notwendige Fundierung der Debatte sorgen. Mit ihrer anwendungsorientierten Forschung könnte die Arbeitswissenschaft verstärkt zum Ziel der Ratsschlussfolgerung vom 5. Dezember 2019 beitragen – den Schutz aller Beschäftigten zu verbessern und dabei insbesondere Beschäftigte in atypischen Beschäftigungsverhältnissen zu berücksichtigen (Council of the European Union 2019, S. 7). Nicht zuletzt geht es dabei auch darum, verbesserte wissenschaftliche Grundlagen vorzulegen, um die Bestimmungen des Arbeits- und Gesundheitsschutzes auch für vulnerable Beschäftigtengruppen, insbesondere in Sub- und Leiharbeitsunternehmen und für Beschäftigte aus anderen europäischen Ländern (Saisonarbeiter*innen, Leih-arbeiter*innen etc.) durchzusetzen.

### Prävention arbeitsbedingter Erkrankungen

Arbeitsbedingte Gesundheitsrisiken und Erkrankungen beeinträchtigen die physische und psychische Gesundheit der Beschäftigten und erschweren, reduzieren oder verhindern die Teilhabe am Arbeitsleben. Die noch immer hohe Verbreitung arbeitsbedingter Erkrankungen in Europa geht zum Teil mit hohen Kosten für Unternehmen und Gesellschaft einher.

Besonders Muskel-Skelett-Belastungen (MSB) nehmen nach wie vor unter der den arbeitsbedingten Gesundheitsrisiken eine unrühmliche Spitzenposition ein. In der Europäischen Union sind sie das häufigste Gesundheitsproblem bei der Arbeit. Ungefähr 60 % der Beschäftigten aus allen Sektoren und Berufen sind von entsprechenden Beschwerden betroffen. Zwischen den Mitgliedstaaten gibt es allerdings große Unterschiede. Während etwa aus Finnland 79 % der Befragten des 6. European Working Conditions Survey über Muskel-Skelett-Beschwerden berichten, waren es in Ungarn nur 40 % (EU-OSHA 2019). In Deutschland sind MSE die Diagnosegruppe mit dem höchsten Anteil an Arbeitsunfähigkeitstagen (BMAS und BAuA 2020, S. 135). Es ist davon auszugehen, dass der Wandel der Arbeitswelt zu einer neuen Verteilung der Risiken bei der Arbeit (z. B. aufgrund sitzender Arbeit) führt, einschließlich der Risiken, die aus kombinierten Expositionen entstehen. Vor diesem Hintergrund betonen die EU-Institutionen seit Jahren die große Bedeutung einer kontinuierlichen Präventionsarbeit (Council of the European Union 2019; EU KOM 2014).

Hinsichtlich psychischer Belastungen erweist sich die Gestaltung guter Arbeit in der betrieblichen Praxis als eine komplexe Aufgabe. Aus Sicht von PEROSH gilt es gerade mit Blick auf gesichertes Gestaltungswissen Forschungslücken zu füllen (Rothe et al. 2017) und durch länderübergreifende Vergleiche von anderen Mitgliedstaaten zu lernen. Es bedarf zum Beispiel eines systematischen, ganzheitlichen betrieblichen Prozesses, in den die betrieblichen Akteure und Beschäftigten einbezogen werden müssen. Insbesondere hierzu kann die Arbeitswissenschaft mit ihrer breiten interdisziplinären Aufstellung verstärkt beitragen.

Zur Prävention arbeitsbedingter Erkrankungen sollte sich die arbeitswissenschaftliche Forschung im engen Schulterschluss mit der Arbeitsmedizin verstärkt dem Ziel widmen, die Auswirkungen des Wandels der Arbeit auf Beschäftigte zu untersuchen und Konzepte für die Primär‑, Sekundär- und Tertiärprävention evidenzbasiert weiterzuentwickeln und zu erproben. Dabei gilt es verstärkt ganzheitliche Perspektiven auf die Gesundheit der Beschäftigten zu fördern und deren Nutzen zu evaluieren.

### Arbeitssicherheit und Gesundheitsschutz entlang von Lieferketten als Probleme der Globalisierung

Globale Lieferketten (Global Supply Chains (GSC)) sind zu einem zentralen Merkmal von Produktion und Handel in der Weltwirtschaft geworden. Im internationalen Vergleich ist die Beteiligung der EU-Länder an globalen Lieferketten besonders hoch (ILO 2016, S. 14). Während GSC zur Beschäftigung beitragen, ist die Einhaltung von Mindeststandards für menschenwürdige Arbeit, einschließlich des Arbeitsschutzes, aufgrund des hohen ökonomischen Drucks, der komplexen Strukturen und des Mangels an verbindlichen Regelungen und deren Durchsetzung oft unzureichend. Seit vielen Jahren versuchen internationale Organisationen, nationale Regierungen sowie Initiativen des privaten Sektors, diese Nachteile von GSC anzugehen. Sowohl die Vereinten Nationen als auch die ILO haben Erklärungen verabschiedet, die Leitprinzipien der Sorgfaltspflicht einführen, um Menschenrechtsverletzungen im Zusammenhang mit globalen Geschäftsaktivitäten zu verhindern (UN 2011; ILO 2017a). Wie eine aktuelle Studie der EU-Kommission zeigt, werden Sorgfaltsstandards zwar teilweise auch von europäischen Unternehmen umgesetzt. Es ist jedoch nicht zu erwarten, dass solche freiwilligen Aktivitäten eine starke soziale Wirkung entfalten (EU KOM 2020c). Viele Arbeitnehmerinnen und Arbeitnehmer vor allem in Entwicklungsländern sind immer noch mit extrem schlechten Arbeitsbedingungen wie Zwangsarbeit, Kinderarbeit, niedrigen Löhne, überlangen Arbeitszeiten, einem hohen Risiko von Arbeitsunfällen etc. konfrontiert. Das Europäische Parlament hat wiederholt auf die Notwendigkeit eines stärkeren EU-Rechtsrahmens zum Schutz der Arbeitnehmerinnen und Arbeitnehmer weltweit hingewiesen, jüngst in einem Votum für eine entsprechende gesetzliche Regelung (Europäisches Parlament 2020). Auch die EU-Kommission kündigte im April 2020 an, im Jahr 2021 eine Gesetzesinitiative zu verpflichtenden Standards für EU-Unternehmen mit Blick auf Menschenrechte und ökologische Sorgfaltspflicht zu starten. Mit der Ausrichtung einer hochrangigen Konferenz zum Thema „Menschenrechte und menschenwürdige Arbeit in globalen Lieferketten“ im Oktober 2020 hat die deutsche Ratspräsidentschaft dieses Thema ebenfalls auf die europäische Agenda gesetzt.

Aus der Perspektive von PEROSH besteht Bedarf sowohl an einer soliden Datenbasis zum Arbeitsschutz in globalen Lieferketten als auch an wissenschaftsbasierten Instrumenten und deren Wirksamkeit, die die europäischen Unternehmen in ihren Bemühungen um die Einhaltung von Arbeitsschutzstandards entlang ihrer Lieferketten praktisch unterstützen. Ein Forschungsprojekt der ILO in Zusammenarbeit mit der Generaldirektion Beschäftigung der EU-Kommission zum Thema Arbeitsschutz in globalen Lieferketten der Lebensmittel- und Landwirtschaft hat wichtige Erkenntnisse erbracht, auf denen in den nächsten Jahren aufgebaut werden kann (ILO 2017b).

### Wirksame Arbeitsschutzsysteme in einer Arbeitswelt im Wandel

Die mit dem Wandel der Arbeitswelt verbundenen Flexibilisierungsprozesse führen zu einer voranschreitenden Herauslösung der Arbeit aus festen räumlichen und zeitlichen Strukturen. Sie werden von einer wachsenden Anzahl von Unternehmen und Beschäftigten genutzt (Sommer 2018). Damit reagieren Unternehmen zum einen auf die Erfordernisse des Marktes bzw. der Kundinnen und Kunden, individuelle Wünsche kurzfristig zu erfüllen, und zum anderen auf die Wünsche der Beschäftigten, private Anforderungen mit denen der Arbeitswelt besser in Einklang zu bringen.

Aus Sicht von PEROSH stellt sich die Frage, wie gesundes und sicheres Arbeiten in neuen Zeitregimes und an wechselnden Arbeitsplätzen gewährleistet werden kann, wenn der Zugang nicht mehr mit den herkömmlichen Methoden, beispielsweise des staatlichen Aufsichtshandelns, gewährleistet ist. Hier ist die Arbeitswissenschaft mit ihrer Expertise gefragt, um zu einer vorausschauenden und menschengerechten Arbeitsgestaltung in zunehmend vernetzten, digitalen und flexibilisierten Arbeitsformen beizutragen. Gleichzeitig gilt es, Arbeitsschutzsysteme im Hinblick auf diese Anforderungen und Gestaltungsmöglichkeiten unter Berücksichtigung der Erfahrungen anderer Länder in Europa als Ausgangspunkt für eine globale Vorgehensweise weiterzuentwickeln. In diesem Sinne könnte eine arbeitswissenschaftliche Forschung zur Wirksamkeit von Arbeitsschutzsystemen und zur Umsetzung auch in solchen neuen Arbeits- und Beschäftigungsformen beitragen (Sommer und Wiencke 2015).

## Fazit

Die letzten Schlussfolgerungen des Rates der Europäischen Union und Stellungnahmen zentraler Beratungsgremien (z. B. des Senior Labour Inspectors Committee (SLIC) oder des Advisory Committee on Safety and Health at Work (ACSH)) stimmen zuversichtlich, dass die vom Forschungsnetzwerk PEROSH erläuterten und in diesem Beitrag grob skizzierten Handlungsfelder für die angewandte Forschung zur Sicherheit und Gesundheit bei der Arbeit Eingang in die neue Arbeitsschutzstrategie der EU-Kommission finden werden. Umso wichtiger ist das Engagement der Arbeitswissenschaft, das europäische Arbeitsschutzhandeln in den kommenden Jahren systematisch zu begleiten und mit wissenschaftlicher Expertise zu stärken. Die skizzierten Herausforderungen sind daher auch von strategischer Relevanz: Sie verdeutlichen die Potenziale einer anwendungsorientierten Arbeitsforschung über die Klärung wissenschaftlicher Detailfragen hinaus. Das aktive Einnehmen einer europäischen Perspektive durch die Arbeitswissenschaft, insbesondere durch den Anschluss an eine europäische Forschungsprogrammatik könnte damit auf gleich zweifache Weise Wirkung entfalten: Neben der Stärkung des europäischen Arbeitsschutzes entstünden auch neue Impulse für die Arbeitswissenschaft – auch für deren globalere Ausrichtung.
